# Aneusomy detection with Karyolite-Bac on Beads® is a cost-efficient and high throughput strategy in the molecular analyses of the early pregnancy conception losses

**DOI:** 10.1186/s13039-015-0168-x

**Published:** 2015-08-12

**Authors:** Javier Pérez-Durán, Zenyese Nájera, Yanelly Trujillo-Cabrera, Mónica Martín-Saro, Ethel García-Latorre, Jaime Escarcega-Preciado, Nayelli Nájera, Teresa Martínez-Galaviz, Gloria Queipo

**Affiliations:** Doctorado en Ciencias Quimicobiológicas, Escuela Nacional de Ciencias Biológicas, Instituto Politécnico Nacional, México City, Mexico; Nanolab Genetic Center, Molecular Biology Laboratory, México City, Mexico; Human Genetic Department, Hospital Materno Infantil ISSEMyM, Estado de México, Mexico; Laboratorio de Inmunoquímica I, Departamento de Inmunología, Escuela Nacional de Ciencias Biológicas, Instituto Politécnico Nacional, México City, Mexico; IECH, Fertility Center, Chihuahua, Chihuahua Mexico; Human Genetics Department, Hospital General de Mexico, Facultad de Medicina Universidad Nacional Autonoma de Mexico, Dr. Balmis 142 Col, Doctores CP, 06766 Mexico City, Mexico DF Mexico

## Abstract

**Background:**

Approximately 10–15 % of all clinically recognized pregnancies end in miscarriage, the majority of them occur during the first trimester, underlying the cause of the loss. Genetic analysis of fetal tissues has the potential to provide valuable information and is highly recommended in some cases. Around 3–4 years ago, the gold standard for the analysis was the GTG Kayrotype, is well known that around 50 % of the tissue samples received failed to grow in culture. Different molecular techniques are used to improve the quality and the specificity of the study, intending to circumvent the limits of the Karyotype.

**Results:**

Karyolite-BoBs™ (KL-BoB™) assay is a recent bead-based suspension, low density array technology with consistent results, probed that is an efficient molecular method to detect aneusomies in early pregnancy losses. Fifty samples from abortions were analyzed in order to probe and give more information about the methodology and analyze if KL-BoBs™ is a good and cost-efficient strategy. We detected 32 % of chromosomal abnormalities, in some of the cases more than one aberration was identified, the array CGH validate the observations.

**Conclusions:**

This molecular strategy is a cost-effective sensitive tool in the early pregnancy loss study.

## Resumen

**Antecedentes:**

Aproximadamente entre el 10–15 % de los embarazos reconocidos clínicamente terminan en aborto involuntario. El análisis genético de los tejidos de aborto proporciona información para el seguimiento médico de algunos casos. El estándar de oro hasta hace pocos años para el análisis de restos embrionarios era el Cariotipo GTG; sin embargo, alrededor del 50 % de las muestras no logran crecer en cultivo, por lo que recientemente se ha implementado el uso de las técnicas moleculares. La plataforma Karyolite-BoBs™ es un microarreglo de baja densidad que analiza con sondas específicas los brazos p, q y centrómeros de los 24 cromosomas los que permite mejorar la calidad y la especificidad del diagnóstico.

**Resultados:**

Se analizaron 50 muestras de abortos espontáneos procedentes de pacientes con pérdidas gestacionales recurrentes provenientes de clínicas de reproducción, mediante Karyolite-BoBsTM, con el objetivo de obtener información del método de análisis y determinar si es una estrategia eficiente con un buen costo-beneficio. Los resultados se validaron mediante array CGH Constitutional Chip R 4.0 microarray de PerkinElmer Resultados: En todos los casos se pudo realizar el estudio molecular. Se detectaron 32 % de anomalías cromosómicas, se observaron alteraciones frecuentes en abortos espontáneos como las trisomías de los cromosomas 18, 21 y la monosomía del cromosoma X. También se identificaron alteraciones poco frecuentes como la monosomía de los cromosomas 11 y 19. En un par de casos se observó más de una aberración. Los resultados se validaron mediante el array CGH y con ambas técnicas se obtuvieron los mismos resultados.

**Conclusiones:**

El análisis molecular mediante Karyolite-BoBsTM es una herramienta sensible y eficaz en costos para el estudio de tejidos embrionarios de pérdida temprana del embarazo.

## Background

Approximately 10–15 % of all clinically recognized pregnancies end in miscarriage. The majority of the products of conception (POC) losses occur during the first trimester of the gestation, before 20 weeks of gestation. Well-known studies suggest that around 50 % of the losses are due to fetal chromosomal abnormalities [1–4].

Genetic analysis of fetal tissues has the potential to provide valuable information regarding the underlying cause of miscarriages, allowing a decrement in the cost of further analysis and genetic counseling of a subsequent recurrence risk. Information on sporadic abnormality, as well as on the presence of a structural chromosomal error associated with a substantial risk for the parents, should be provided to the patients. Determining the genetic cause of the reproduction failure is an important issue for couples who want a healthy pregnancy; the obtained information has an impact on the family’s plans and future management. It has been observed that most couples feel relieved when they know the cause of the pregnancy loss [4–6].

Most of the time, these kinds of studies are part of a protocol for reproductive problems. Until 3 to 4 years ago, the gold standard for genetic analysis was the tissue culture and chromosomal GTG karyotype. Consistent evidence in the literature shows that the tissue culture has many limitations for many reasons, including the way that POC is obtained, if the sample needs to be translated to the cytogenetic laboratory, or if the tissue, per se, has a great potential of contamination. The amount of embryonic material, most of the time is not enough to obtain reliable results. The literature indicates that around 50 % of the tissue samples received failed to grow in culture [7]. In the remaining cases, the slow cell growth from the time of the receipt the sample and report is around one month. It is well known that conventional cytogenetic techniques depend on skilled technicians; and maternal contamination is not rare, causing errors in the results like false normal female. For all the reasons mentioned above, it is considered that the conventional cytogenetic is a not-efficient or cost-efficient, low-throughput study (Table 1) [5]. The solution to all these problems is the use of molecular techniques. Different strategies have been used in order to improve the finding of chromosomal abnormalities, trying to circumvent the limits of the cytogenetic analyses [8–10]. Our laboratory has had a positive experience with the use of the recent bead-based suspension, low-density array technology, bacterial chromosome (BAC) on beads (PerkinElmer, Wallac Oy, Turku, Finland). Two previous papers mention the utility of this strategy in the POC analysis; our laboratory evaluated and probed the feasibility in the abortion tissue analysis using the Karyolite-BACs on Beads (KL-BoBs™) [11–14], which provides low resolution coverage of all chromosomes. Demonstrating this is a good and cost-efficient strategy that could be used in countries with low economical resources. In this report, we analyzed 50 consecutive POCs with KL-BoBs™.

**Table 1:** KL-BOBs™ and conventional cytogenetic characteristics

	KL-BOBs™	GTG Karyotype
Tissue Culture	Not necessary	Necessary, 50 % fails to grow
Final result	24–48 h	1 month
Interpretation of the results	Friendly-software	Skilled and trained lab-person
Throughput-technique	High	Low
Technical problems	DNA Extraction (very rare), maternal contamination	Bacterial infection, maternal cell contamination, tissue maceration, poor chromosomal morphology
Chromosomal abnormalities detection	Aneuploides (representing 90 % of the pregnancy losses)	Numerical, structural, triploidies and tetraploidies
Mosaics	Above 15–20 %	At leaste25 good quality metaphases
Cost efficient	Cost efficient	Not-cost efficient

Advantages and disadvantages of each technique

## Results

**Fig. 1:**
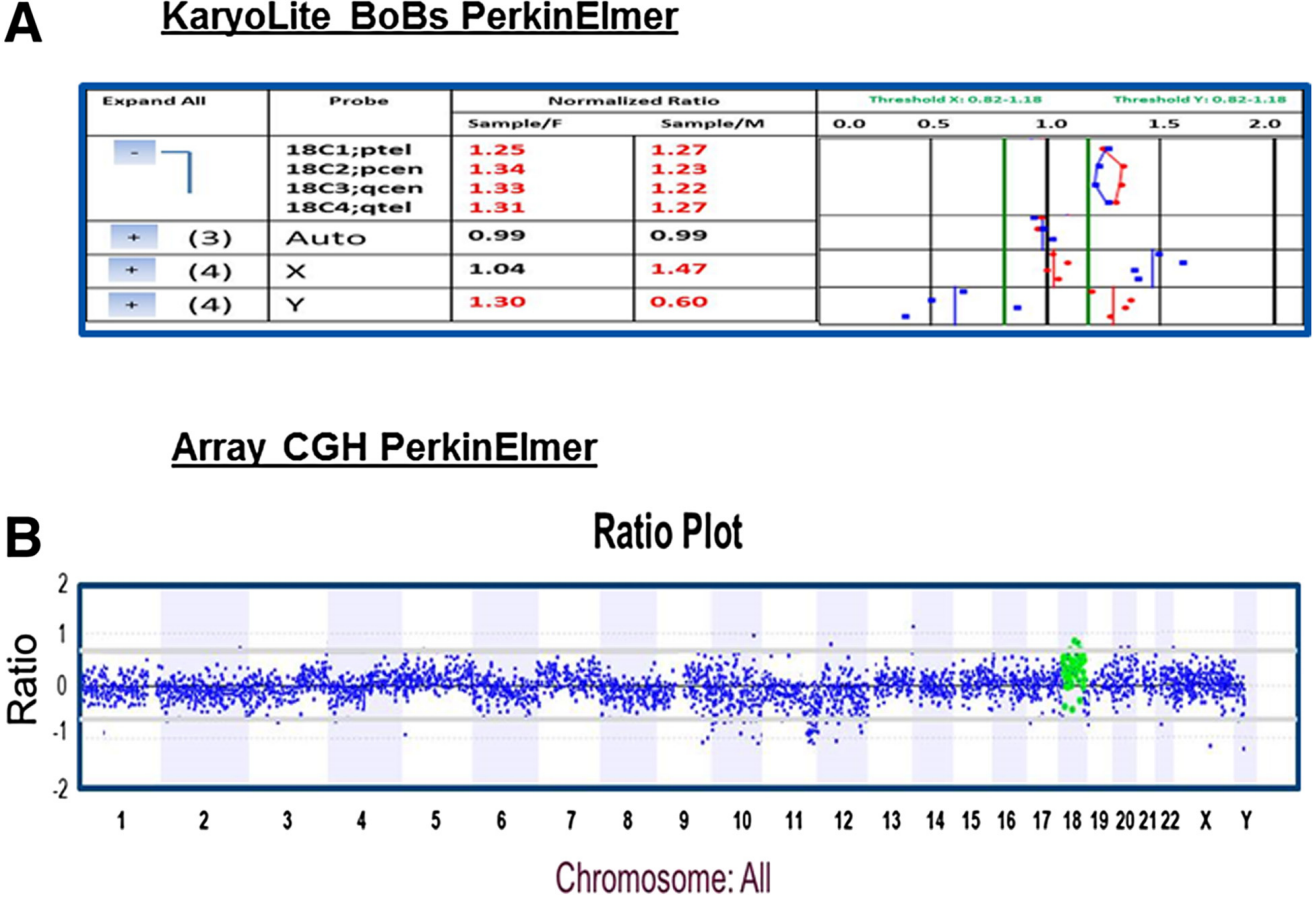
Cases whit trisomy 18. **a** Karyolite BoBs female with gain in chromosome 18. **b** Trisomy of chromosome 18 was confirmed by array comparative genomic hybridization whit a Constitutional Chip^R^ 4.0^1^ microarray, PerkinElmer

**Fig. 2:**
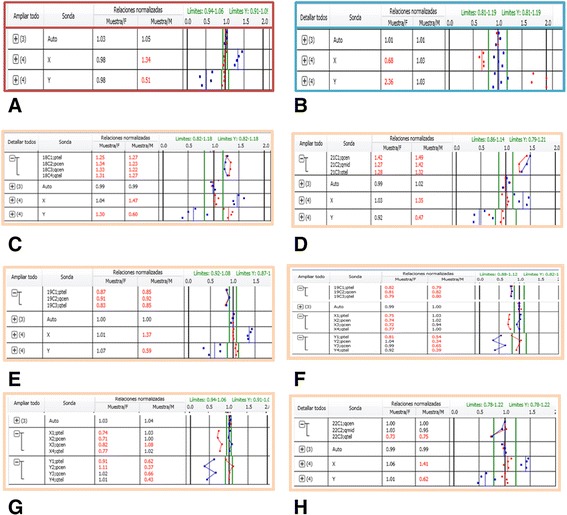
Examples of KL-BoBs™ results, samples with normal and abnormal results: **a** Normal female (46, XX); **b** Normal male (46, XY); **c** Female whit gain of chromosome 18 (47,XX +18); **d** Female whit gain of chromosome 21 (47,XX +21); **e** Female whit loss of chromosome 19 (45,XX −19); **f** Turner with a loss of chromosome 19 (44, X0 −19); **g** Turner (45, X0); **h** Female whit a loss of 22qtel probe (46,XX – 22qtel)

KL-BoBs™ was performed in 50 consecutive PCO samples; all of them were spontaneous, early gestational losses. The tissue samples were recovered from different centers and obtaining heterogeneous samples. All the collected samples were from the first trimester, all were considered recurrent miscarriages, and all were part of an assisted reproductive program. In 20 of them, it was difficult to reach the quality of the sample in the laboratory; some of the centers sent large amounts of tissue, including the gestational sac. However, in all the cases, the DNA quality was optimums for performing the assay. The KL-BoBs™ identified 32 % of chromosomal abnormalities (Table 2). Half of them were as expected, the most frequent chromosomal abnormalities like Down and Turner syndromes and one trisomy 13. The rest of the findings were rare, including, two monosomies and one trisomy on the chromosome 19, one monosomy of chromosome 14, and one trisomy of chromosome 11 (Fig. 1). In two cases, a double alteration was identified. In order to corroborate the results, we validated some of the results with an array CGH (aCGH) form PerkinElmer Company. The results obtained with the aCGH were the same as the ones observed with the KL-BoBs™ (Fig. 2). The results without alterations in the regions analyzed were 10 cases 46, XY and 24 cases 46, XX. In the 46, XY PCOs the abnormalities were around 50 % while in the 46, XX were about 19 %.

**Table 2:** Summarize the results of all the PCOs analyzed KaryoLite-BoBs™ technology

Normal		34 samples
	Chromosome complement	Cases
	46,XX	24
	46,XY	10
Anormal		16 samples
	Chromosome complement	Cases
	XY +21	3
	45, X0	3
	XX −19	2
	XY +11	1
	XY +13	1
	XY,+19	1
	XX −14	1
	X0 −19	1
	46,XY +21 qcen	1
	46,XX del 22qter	1
	XY, +19 ptel+19 q cen+22q tel	1

## Discussion

There is no doubt about the tissue culture limitations in the study of the PCOs. The molecular techniques are the most reliable strategies for detecting chromosomal abnormalities in the PCO tissue samples (Table 1). After a positive experience with the prenatal BOBs™, we decided to analyze a consecutive series of 50 PCOs using the KL-BoBs™ assay in order to consider this strategy an affordable and useful strategy for the detection of chromosomal abnormalities in the abortion samples, and give more information about the technology and its limitations in our population. We performed the study in samples from recurrent early pregnancy losses before 12 weeks of gestation. The literature and our experience in the laboratory estimated that around 50 % of the samples fail to grow [1, 7, 10]. Around 90 % of the chromosomal findings are due to numerical abnormalities, 6 % from structural abnormalities, and the remainder from anomalies such as mosaicism. Chromosomal abnormality has been found to have a direct correlation with gestational age. Balanced reciprocal translocations are relatively common, with an incidence around 1:500. Conventional cytogenetic detects this anomaly, however, balanced reciprocal translocations do not cause PCO. Is important to mention that KL-Bobs™ have the potential to detect the unbalanced progeny of balanced carriers with a reciprocal translocation [15, 16]. The time to obtain a report takes a month and depends on the cytogenetic technician’s skills, and the study resolution is around 10 to 15 MB. The main point is that conventional cytogenetics is not a cost-efficient and low-throughput study [5]. Different molecular techniques are used to improve the quality and the specificity of the study, intending to circumvent the limits of the GTG Karyotype. Targeted molecular methods such as multiplex ligation mediated amplification, quantitative fluorescence PCR (QF-PCR), and fluorescence in situ hybridization have been successfully applied to POCs [5, 6]. These methods are also less sensitive than chromosomal microarrays because of their low multiplexing capabilities. On the other hand, performing aCGH is still expensive and is not possible in all cases. The development of a molecular strategy based on microarray technology is a good strategy for the PCO analyses. Three previous reports using a novel strategy with KL-BoBs™ demonstrated that it is a useful strategy for detecting chromosomal imbalances [13, 17, 18]. Our experience observed 32 % with abnormalities, which we considered to be a low percentage, as expected. We supposed that this is because of the maternal contamination in the normal 46,XX females. This observation was confirmed when we analyzed separately the XY results, obtaining 50 % with abnormalities, as was expected and reported in the literature. Chromosome abnormalities have been recognized to be the major factor contributing to pregnancy loss before 20 weeks of gestation (wg), accounting for 32 to 72 % of all miscarriages [4, 7]. Considering that maternal contamination is one of the disadvantages of this technique, this issue could be fixed with a correct sample reaching; in our case, the large amount of tissue sent to the lab was the cause of this data. Nevertheless, the maternal contamination is worse in the tissue culture because the overgrowth of the maternal cells over the embryonic or fetal cells increases the false negative results [19, 20].

After this study, we are teaching the personnel to take only 0.5 cm of the embryonic or embryonic sac tissue sample, no more, and put it in a 2 mL eppendorf collection tube. Paxton et al., after studying 3794 consecutive clinical cases, discussed that, with a perfect performance of the assay, as many as 90 % of all PCOs could be correctly classified by this assay when compared with the karyotype [20]. This could be a distinctive advantage in terms of more rapid results, elimination of the tissue culture, and the fact that it is a low-cost assay compared with the microarrays. One of the major limitations of the KL-BoBs ™ technique is that it cannot detect triploid, tetraploid, or structural abnormalities, which, together, comprise about 10 % of the total of PCOs etiology. One report in the literature demonstrated the ability of this technique to detect male prenatal triploidy [11]. In our lab, at least 200 prenatal tests have been done and this phenomenon has never been observed. The assay could detect mosaics when they have more than 20–30 % of the cells; so far, this is a disadvantage if there is a low percentage mosaic. Previous reports showed that the findings observed by the KL-BoBs™ were corroborated by aCGH, in which the array was done in 99/100 samples with Single Nucleotide Polymorphism (SNP) 6.0 microarray; results of the KL-BoBs™ assay were 100 % concordant with the microarray for all the aneusomies [21]. Nevertheless, the microarray has a higher resolution while KL-BoBs™ has a very high sensitivity for aneusomies detection. The aCHG that we performed in some of our samples corroborated the diagnosis performed with the KL-BoBs™. In our series, we found more than one aneusomie, which is not an unusual finding because the technology is capable to detect it. In a previous series, the authors found that around 74 % of the PCOs have more than one aneusomie. These observations are quite difficult to observe with conventional karyotypes, while KL-BoBs™ and the microarray are good strategies for detecting them.

In conclusion, the use of KL-BoBs™ is a sensitive molecular tool to analyze the 46 chromosomes in PCOs, and detect aneusomies with a cost-effective study. SNPs and CGH array analysis are expensive, while this molecular analysis performs a successful low-density array for the 46 chromosomes.

## Methods

Fifty POCs were included in the study and all samples were single pregnancies. Samples were collected in a saline solution and transported at room temperature. Purified DNA was extracted directly from residual cleaning, using QIAamp DNA Mini Kit extraction reagents for tissues (Qiagen, USA). The quality and the amount of the DNA were analyzed with a nanodrop instrument.

### KaryoLite-BoBs™

**Fig. 3:**
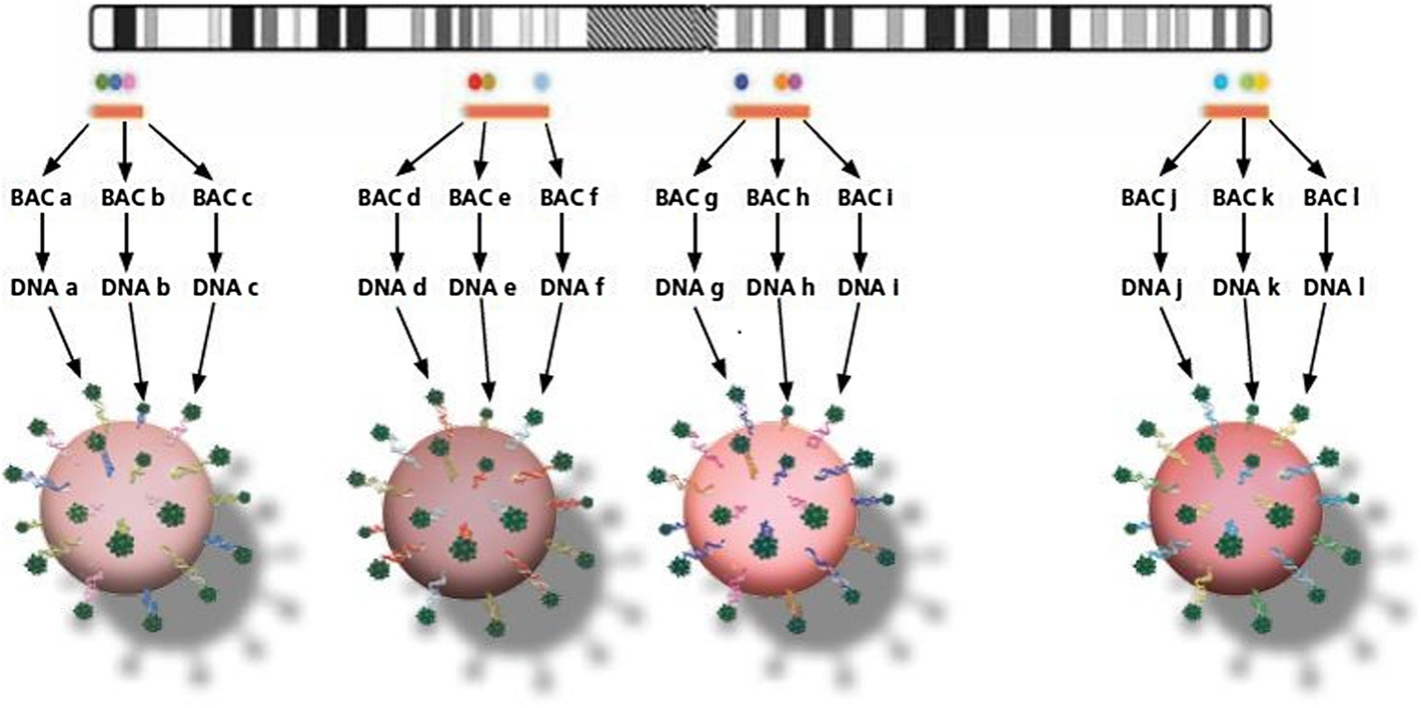
KaryoLite™ BoBs™ assay design. Each bead is a composite of multiple neighboring bacterial artificial chromosomes according to the Human Genome Build GRCh37.2/hg19 assembly. For each chromosome, except the acrocentric ones, two beads per arm were used, providing dosage information on telomeric and pericentromeric regions. Figure from PerkinElmer educational material

The microarray KL-BoBs™ was performed with 240 ng of genomic DNA from abortion tissues. The assay provides dosage information about the proximal and terminal regions of each chromosome arm by using beads: 91 beads are a composite of three neighboring BACs in equal amounts, according to the Human Genome Build (GRCh37.2/hg19) assembly, which detects arm-specific aneuploidies in all 24 chromosomes in a single assay. The microarray includes beads composed of three BACs and covers centromere, p and q arms of all chromosomes (1–22, X and Y) (Fig. 3). The product covers q arms in acrocentric chromosomes. BACs-on-Beads technology consists of BAC DNA immobilized into polystyrene microspheres distinguishable by the Luminex® instrument system. The assay utilizes probes immobilized on polystyrene microspheres (beads) and impregnated with a specific ratio of red to infrared dyes to allow bead identification (Fig. 3).

The procedure is summarized in four steps:

1. Labeling of genomic DNA. Purified whole genome DNA from the sample, male and female controls (Promega, Madison, WI) 240 ng was linearly amplified with a biotin-labeled dNTPs mix at 37 °C for 60 min.
2. Purification of labeled DNA. Labeled DNA was purified with the PureLink™ PCR Purification Kit (Invitrogen/Life Technologies, CA) to remove un-hybridized biotin-labeled dNTPs. DNA was quantified with a nanodrop and diluted to 150 to 200 ng/μL.
3. DNA hybridization. Biotin-labeled samples and controls were hybridized to the Karyolite-BoBs™ bead set in a TriNest™ shaking incubator (PerkinElmer, Turku, Finland) at 52 °C to 1200 rpm for 16 h.
4. DNA washing and reporter binding. The reaction was stopped with a wash buffer and shaken at 50 °C for 20 more minutes. The biotin-labeled DNA hybridized was revealed with streptavidin phycoerythrin for 30 min at 37 °C.

Samples were washed and run on a Luminex100–200™. The Luminex technology employs a red–infrared laser to identify the region of each probe and measures the relative amount of biotin-labeled DNA hybridized to each bead with a green laser. The aneusomy analysis was performed using a software program, BoBsoft™ v1.1, PerkinElmer, Waltham, MA. In BoBsoft™.

**Fig. 4:**
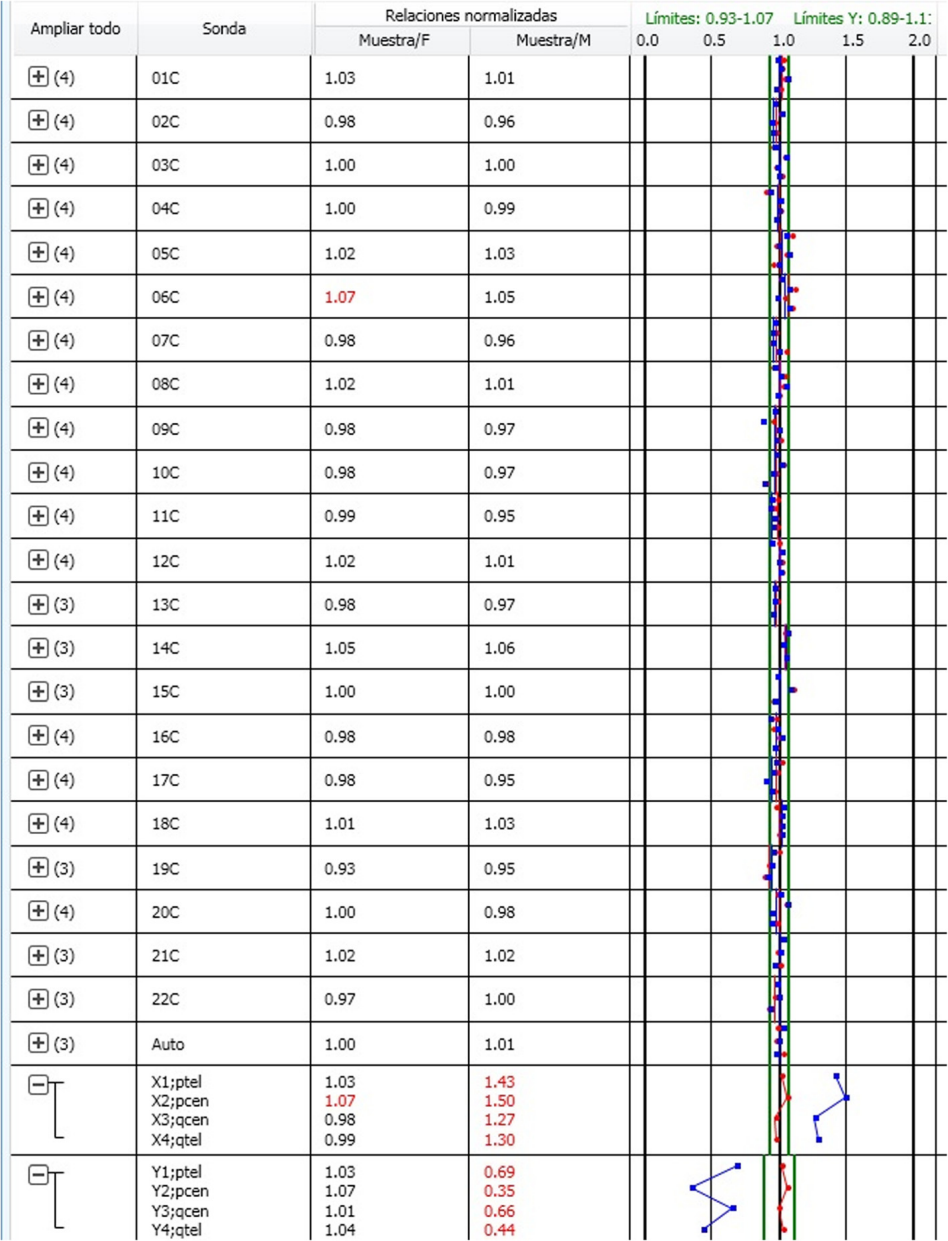
Representative KaryoLite-BoBs^™^, result from the normal female 46, XX. The Graphically plot shows the hybridization intensity of each probe set, in the 24chomosomes, in relation to the female (*red*) and male (*blue*) reference standards

Aneusomy detection with the KL-BoBs™ assay was performed by comparing the test sample on four reference DNA samples (two female and two male; Promega, Madison, WI). The Luminex .csv data file was imported and the data was analyzed using a software program (BoBsoft™ v1.1, PerkinElmer, Waltham, MA). In BoBsoft™, chromosome enumeration is presented numerically as a sample to reference ratios for each probe set (i.e. bead), and graphically as a plot, showing the hybridization intensity of each probe set in relation to the female (red) and male (blue) reference standards (Fig. 4).

### Constitutional Chip ^R^ 4.0 microarray

The constitutional Chip 4.0 is comprised of BAC (Bacterial Artificial Chromosome) clones containing large segments (~100–300 kb) of human DNA. With approximately 5000 clones, the constitutional Chip 4.0 covers the whole human genome with an average resolution of ˂650 kb, the array is high resolution tool for detecting gains or losses of DNA in chromosomes. Two 2 μg of purified DNA were required for analysis. Labelling and hybridization of test and reference DNA were performed according to the manufacturer’s protocols. Test and reference DNA were labelled by Cy3 and Cy5 on two independent labeling reactions, with reciprocal labelling reactions, and were hybridized for 16 h. Arrays were washed four times according to the protocol. Two color scans were performed by the PerkinElmer® Inc.’s ScanArray® Microarray Scanner. Data extraction, analysis, and visualization were done by the Oneclick CGH software, PerkinElmer Edition. Data from the hybridization experiment was represented as a ratio of the two signals (Cyanine 3/Cyanine 5) and normalized to correct for non-biological effects in the data.
